# mSphere of Influence: Lighting up organellar communication in protozoan parasites

**DOI:** 10.1128/msphere.00574-24

**Published:** 2025-02-06

**Authors:** Diego Huet

**Affiliations:** 1 Department of Pharmaceutical and Biomedical Sciences, University of Georgia, Athens, Georgia, USA; 2 Center for Tropical and Emerging Global Diseases, University of Georgia, Athens, Georgia, USA; University of Georgia, Athens, Georgia, USA

**Keywords:** *Toxoplasma*, organelles, proximity biotinylation

## Abstract

Diego Huet works in molecular parasitology, focusing on the organellar biology of *Toxoplasma gondii*. In this mSphere of Influence article, he reflects on how the article “Efficient proximity labeling in living cells and organisms with turboID” (Branon et al., 2018) impacted his research and the strategies used to dissect inter-organellar interactions in *T. gondii*.

## COMMENTARY

Within the eukaryotic cell, effective communication between organelles is crucial for carrying out essential cellular processes. It is now well established that organelles interact with each other via membrane contact sites (MCSs) in addition to vesicular trafficking or cytoplasmic diffusion (1). Although inter-organellar communication is a ubiquitous phenomenon across eukaryotic cells, the functional significance and molecular composition of MCSs have only been extensively characterized in yeast, plants, and mammals. These three groups constitute only a small fraction of eukaryotic diversity, so what about the rest? What about protists, which encompass the majority of this diversity? The study of MCSs in protists—many of which harbor phylum-specific organelles—is in its nascent stages (2–4), and my laboratory is actively studying them in *Toxoplasma gondii*, a parasitic protist.

How does one investigate MCSs? Proximity biotinylation is a technique that has advanced our understanding of these structures—albeit only in a handful of organisms as I mentioned before. It is based on the use of a promiscuous biotin ligase to label proteins in a proximity-dependent manner upon biotin addition (5). Labeled proteins can subsequently be isolated via immunoprecipitation using streptavidin beads and subjected to mass spectrometry analysis. Additionally, proximity biotinylation does not require any pre-existing knowledge of the MCS composition, a benefit when you study *T. gondii* or any non-conventional model organism where the vast majority of the protein-coding genes remain uncharacterized. When thinking about ways to study MCSs in *T. gondii*, I stumbled upon the article by Branon and colleagues (6). Branon et al. used directed evolution to generate TurboID, a new version of the promiscuous biotin ligase BirA. TurboID biotinylated other proteins much faster than the original BirA, taking only 10 min compared with 18 h. Importantly, Branon et al. demonstrated the feasibility of using TurboID to rapidly label different cellular compartments, including the mitochondrial matrix and the cytosolic face of the endoplasmic reticulum. They used an elegant mass spectrometry-based analysis approach to demonstrate that TurboID can robustly label a substantial portion of the known mitochondrial (67%) and endoplasmic reticulum (ER) (87%) human proteomes. To make their analysis more stringent, they also used a spatial reference control by targeting TurboID to the cytosol. By comparing the proteins labeled by TurboID when it was targeted to an organelle with those labeled when in the cytosol, Branon et al. were able to determine which proteins were specifically associated with a particular organelle.

To summarize, proximity biotinylation using TurboID, and a recent, novel iteration called Split-TurboID, represent a powerful tool for investigating MCSs. Despite the low expression and a potential dynamic localization—including stimulus-dependent recruitment—of many proteins in these microdomains, the use of controls, such as a spatial reference and the subsequent validation of potential candidates, has enabled the identification of novel MCS proteins in mammals (7, 8). Proximity biotinylation has significantly helped my group to develop a strategy to investigate MCSs in *T. gondii* using an unbiased proteomic approach. We have been able to target TurboID to the cytosolic surfaces of the mitochondrion (*T. gondii* has a single mitochondrion), apicoplast (a plant-like organelle only found in *T. gondii* and its relatives), and endoplasmic reticulum. Combined with a spatial reference control (cytosolic TurboID), we are hopeful that the approach developed by Branon and colleagues, which has also been subsequently refined and published as a protocol (9), will help us determine the composition of MCSs in *T. gondii* and provide novel insights into organisms beyond conventional biological models.
